# Why do some physicians choose to tackle inequities in healthcare?

**DOI:** 10.1186/s12939-018-0790-4

**Published:** 2018-06-15

**Authors:** Victoria Nkunu, Kevin J. McLaughlin

**Affiliations:** Cumming School of Medicine, University of Calgary, 3330 Hospital Drive NW, Calgary, AB T2N 4N1 Canada

**Keywords:** Health inequity, Physicians, Realism, Grounded theory

## Abstract

**Background:**

Despite the reputation of Canada’s healthcare system as being accessible to all Canadians, certain populations continue to face inequities within our healthcare system. In addition to promoting fairness, addressing healthcare inequities has the potential to reduce healthcare costs, which is increasingly important as healthcare costs continue to rise. Intentionally or otherwise, physicians are often leaders in healthcare teams, but there is a paucity of literature on physicians’ perceptions of the problem of healthcare inequities and their potential role in addressing inequities. In this pilot study, we use a grounded theory approach to explore contextual factors and mechanisms that associate with an individual physician’s involvement (or otherwise) in initiatives to reduce healthcare inequity.

**Methods:**

Using purposeful sampling and a set of a priori questions, we interviewed ten physicians – five of whom self-identified as being actively involved and five not actively involved in addressing healthcare inequities – to explore potential reasons for physicians choosing to address the causes of healthcare inequities.

**Results:**

We identified contextual barriers (e.g., lack of knowledge and time) and facilitators (prior experience, protected time, mentorship and system supports) that we interpreted as interacting with the underlying mechanism (motivation to address inequities) to influence a physician’s decision on whether or not to address healthcare inequities.

**Conclusion:**

Based upon our findings we propose further studies to understand and/or overcome barriers to physicians being involved in addressing healthcare inequities.

*“As long as poverty, injustice and gross inequality persist in our world, none of us can truly rest.”*

-Nelson Mandela

## Background

The Canadian healthcare system is often regarded as a shining example of a well-structured and accessible healthcare system. But what often gets lost in this portrayal, is the fact that significant sections of the population are unable to access necessary health services, due to many complex barriers. Sadly, this is not breaking news. In a report published in 2011, the Canadian Medical Association (CMA) acknowledged that inequity exists within the healthcare system, and addressing it should be priority for all stakeholders (i.e., the government, patients, and medical professionals) [1]. In the same report, the CMA highlighted the importance of supporting physicians to play a role in addressing this inequity. More recently, the former Minister of Health, Jane Philpott emphasized the role of physicians in addressing health inequity [2]. Intentionally or otherwise, physicians are leaders of healthcare teams that care for patients, which places them in a unique position to observe how health inequities directly impact patient care. However, relatively little is known about Canadian physicians’ understanding of inequity within the healthcare system, and what role they can play in addressing it.

In the United States, where almost 30 million Americans have no health insurance [3], health inequities and barriers to care are hard to miss. In contrast, inequities in the Canadian healthcare system are more insidious owing to the fact that, by law, all Canadian residents should have access to necessary healthcare services without paying out of pocket [4]. But research has shown that this is not the case for many patients. For example, refugees [5], individuals experiencing poverty and homelessness [6], and Indigenous Canadians [7] are disadvantaged when it comes to healthcare delivery.

Should physicians be concerned by inequities in the Canadian healthcare system and try to mitigate these? Philosophically, it is tough to argue that when working in a healthcare system where equity is a driving principle we should simply accept that inequity is inevitable. This moral argument is complemented by the fact that addressing inequities also makes financial sense. For example, Roos et al. estimated that by reducing socio-economic differences in health, 15% of total hospital expenditures could have been saved [8]. But physicians have many competing demands for their time and, although the Canadian Medical Association believes that, as leaders of the healthcare system, physicians can be involved in addressing healthcare inequities, there is no obligation for each individual physician to adopt this role. Thus, the decision on whether and how to become involved in addressing healthcare inequities lies with the individual: some physicians become advocates and champion reform to address inequities, while others choose to direct their attention elsewhere. So, why do some physicians choose to tackle inequities in healthcare?

In this pilot study, we explore contextual factors and mechanisms that are associated with an individual physician’s self-identified involvement in initiatives to reduce healthcare inequity. We predicted that all participating physicians would be aware of inequities in the healthcare system, but would differ in their attitude on whether and how physicians can address healthcare inequities. We used purposeful sampling and included physicians who self-identified as being actively involved in initiatives to reduce healthcare inequities and those who self-identified as not being involved and used the framework of realism [9], to describe contextual factors and mechanisms that are barriers or facilitators to physicians being involved in initiatives to reduce healthcare inequities.

## Methods

### Participants

Participants were physicians from a variety of medical specialties who practised in Southern Alberta, with a majority from within Calgary and surrounding areas. Research participants were recruited through personal contacts and through referrals from other physicians. Using purposeful sampling, a total of 10 physicians were interviewed. Prior to the interview, we asked each participant if they considered themselves to be actively involved in addressing health inequities, or not. By so doing, we were able to purposefully sample 5 who self-identified as being actively involved in initiatives to reduce health inequity (AI) and 5 who self-identified as not being involved (NAI) for interviewing. Any self-identified level of involvement in any initiative to reduce health inequities, in Canada or abroad, was considered as AI for the purposes of our study. Prior to beginning our interviews, we obtained written informed consent from all participants.

### Materials

The materials for this study were transcripts derived from individual interviews with participants. We conducted two practice interviews and, based upon these, created a planned format for our interview that is detailed below in Appendix 1.

### Procedure

We contacted prospective participants and invited them to participate in an individual interview, either in person (*n* = 9) or by conference call (*n* = 1). We used a priori questions (Appendix 1) but also asked further questions for clarification or to explore emerging themes. There were two interviewers: one primarily asking the interview questions (VN), and the other note-taking and asking follow-up questions as needed (KM). We anonymized all responses and referred to participants by study number [1–10]. Once the interviews were completed, we emailed the final manuscript to the interviewees for triangulation and to ensure that they felt that their comments did not reveal their identity.

After each interview, VN and KM analyzed the interview transcript and performed constant comparative analysis of emerging themes. Both VN and KM analyzed the transcripts individually and coded the responses for themes. Afterward, VN and KM compared their codes for agreement. When disagreements arose, VN and KM would discuss until both came to a consensus. Because our sample size was too small to prove thematic saturation, constant comparative analysis allowed us to identify major themes, that we could investigate later with a study of a larger sample size.

### Analysis

Using a grounded theory approach [10], VN and KM analyzed the interview transcripts and performed constant comparative analysis of emerging themes. We used a realist framework to organize these themes into contextual factors and mechanism with the goal of theorizing on why individual physicians choose to become involved in initiatives to address healthcare inequities while others do not.

## Results

Of the 10 participants we interviewed, six identified as being female and four as male; four were geographical full time and six were in full- time clinical practice; four had been in practice for < 10 years, four for 10–20 years, and two for > 20 years.

### Are you aware of inequities in healthcare? How did you become aware of this?

All individuals felt that there are inequities in the healthcare system. Four out of five participants in the NAI group became aware of inequities through their clinical experiences (one commented that “this was not a focus at medical school”), while one NAI participant reported becoming aware of this in their undergraduate curriculum. Of the participants in the AI group, three became aware of health inequities through their experiences in healthcare prior to becoming physicians where they either worked in a healthcare setting close to First Nations reserves or through international volunteer work. Another developed a greater understanding of inequities in volunteer work with the Calgary Student Run Clinic and from contact with physicians who had worked at The Drop-In Centre and other homeless shelters in Calgary. Two individuals in the AI group also reported that, as immigrants to North America, they felt very fortunate to have the opportunity to have further training and had a life-long ambition to “give back” to the system.

*NAI: “…I became aware of healthcare inequities from the news and day-to-day in my practise.”*

*AI: “Prior to my career in medicine, I worked as a nurse in an outpost station, where many patients were from First Nations reserves.”*

### Have you given any thought as to why these inequities exist and how to overcome them?

In the NAI group, some participants felt that centralization of healthcare in “big hospitals with expensive parking” and “social engineering, i.e., where government [chooses to] put its money and services” creates inequities, and that homelessness and financial difficulties fuel these inequities. Participants in the AI group also identified centralization of resources as contributing to inequities. They also highlighted other important contributory factors, such as “social determinants of health” (including education, housing, employment, etc.) and cultural barriers, including “racism among providers”, not having a family doctor and not knowing how to access the healthcare system. One participant in the AI group also felt that the need to provide equity to physicians (e.g., OR time) creates inequities for patients since resources are not necessarily matched to patients’ needs.

*NAI: “…System changes are necessary to improve inefficiency causing inequities, for example big hospitals with expensive parking.”*

*AI: “Many reasons, including not knowing how the system works, not having a GP and being able to book appointments in advance.”*

### Can doctors have a role in addressing these inequities? If yes, how? If no, what are the barriers?

Each NAI participant felt that doctors can have a role, which includes advocating for patients on an individual basis and at an administrative level, for example by lobbying government to “put services where people are”. Those in the AI group agreed that physicians can help at both the individual and administrative levels, but also felt that training institutions can play a role by improving physicians’ understanding of healthcare inequities and addressing cultural perceptions, which could then result in more appropriate role modelling for students and residents.

*NAI: “I think so. Doctors can do something but not everything. Can’t ask them to advocate for everything but they can help in administration.”*

*AI: “Definitely. Doctors are attributed status in society. This makes advocacy so important.”*

### Should doctors have a role? Why or why not?

Each of the participants felt that doctors should have a role. Several participants felt that this was not optional and was part of being a doctor as it was “an ethical and moral responsibility to provide care for all types of patients”. Reasons why physicians should have a role according the participants in the NAI group included the fact that the title of “Doctor” carries weight at both the individual and political level. In the AI group, reasons why physicians should be involved also included the fact that “they are the first line in a crisis situation” and that they have a “unique position through their direct contact with patients.” AI participants also offered that the status of being a doctor afforded an opportunity to become involved at a community and policy-making level.

*NAI: “Yes. Doctors are ethically and morally responsible to promote care for all types of patients”.*

*AI: “Yes. Doctors are in a unique position, [because] they have direct contact with patients experiencing inequities.”*

### Have you been involved in any initiatives, research projects, or other types of work to address a health inequity? What are some factors that enabled you to engage in this type of work (facilitators) or what barriers have you encountered that have made it difficult for you to engage in this type of work?

Those who self-identified as NAI actually did report some involvement on an individual level, such as making telephone calls on behalf of patients and involvement in teaching about healthcare inequities. In the NAI group, barriers included lack of time, not knowing how to report inequities in healthcare, and lack of mentors and role models. In the AI group, facilitators included having financial and administrative support and time to spend on projects to address inequities, having specialized training and/or a practice location that provided frequent access to “marginalized communities”. AI participants, in addition to mentioning similar barriers as the NAI group like lack of time and lack of mentorship, also identified challenges that they needed to overcome, including “gaining the trust of the community”, accessing healthcare records on First Nations reserves, inability to match the available time and resources to the needs of individuals in crisis (e.g., need for a “detox bed”, mental health resources, and housing). The latter can be helped by increased awareness of resources that can be accessed in times of crisis, such a social work services. Several AI participants felt motivated to address healthcare inequities due to the simple fact that if these issues are not addressed then “patients tend to present again with the same problems a few days later”. Interventions that AI participants have been involved in included setting up clinics on First Nations reserves, dissemination of education on the Truth and Reconciliation Call to Action, creating a module on cultural competencies for new healthcare employees, and improving education to physicians and patients on how to access resources in times of crisis.

*NAI: “No. But I try to find a solution by myself, for example doing telephone consults for patients who cannot come to clinic.”*

*AI: “My whole practise revolves around vulnerable individuals, like the homeless, refugees…”.*

### In your perspective, what is one of the greatest health inequities that needs to be addressed?

NAI participants felt that the greatest inequities were around homelessness, lack of a family physician, lack of clean water, lack of education, and inability to access healthcare due to language barrier. AI participants identified financial barriers to accessing healthcare and a combination of high burden of disease and reduced access to services in the local community (particularly on First Nations reserves). Other contributory factors include cultural barriers, lack of physicians from a First Nations background, and systemic discrimination against First Nations peoples, as illustrated by one AI participant who stated that “as a refugee kid, I had a better chance compared to Indigenous kids.”

*NAI: “Global disparities including access to clean water, education…”.*

*AI: “Access to services in local community and burden of disease in First nations communities.”*

#### A realist explanation of why some physicians choose to tackle inequities in healthcare

**Fig. 1:**
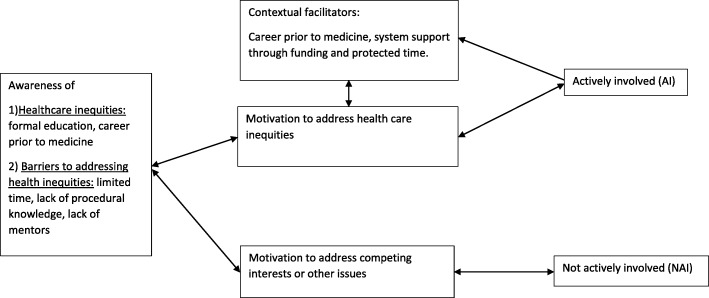
Theoretical relationship between context, mechanism and involvement or non-involvement in initiatives to address health inequities

Given the fact that both groups of participants (NAI and AI) shared awareness of healthcare inequities, the belief that physicians should be involved in addressing inequities, and the experience of encountering barriers to becoming involved – but differed in their choice on whether or not to become actively involved – we felt that a realist approach [11] to explore “what works in which circumstances and for whom?” was appropriate to address our research question: why do some physicians choose to tackle inequities in healthcare? Figure 1 shows our interpretation of the relationship between the themes that emerged from the interviews (Table 1).

**Table 1:** Codes and illustrative comments from interviews

Code	Illustrative comments
↑awareness through formal education	*“…As an undergraduate student, I majored in cultural anthropology… involved in social justice, to address disparities and inequities.”* *“…I became aware of inequities during my undergraduate medical education and through clinical experience…”*
↑awareness through career prior to medicine	*“…Prior to my career in medicine, I worked as a nurse in an outpost station…where many patients were from First Nations reserves.”* *“…Before I started medical school, I volunteered in developing countries, where I saw that poverty leads to bad health.”*
limited time	*“…My biggest barrier is lack of time and lack of mentors”* *“… I have done an elective to increase exposure to vulnerable populations, but ultimately a lack of time is a barrier…”* *“… lack of time to advocate for individual patients…”*
lack of procedural knowledge	*“… No one to tell about parking cost affecting patients, so I try to find a solution myself … but can’t do that for every patient.”*
lack of mentorship	*“… besides lack of time, I am not aware of what others are doing.”* *“… I don’t know what [projects are] going on…”* *“…My biggest barrier is lack of time and lack of mentors”*
system support	*“… the Dean is supportive of my work.”* *“… the administration is supportive of my volunteer work.”* *“…and funding from [the University] to enable me to travel and work…”* *“…I am in a unique position to practice medicine and work with the AHS (Alberta Health Services) to advocate for Indigenous health.”*

In a healthcare system where physicians are afforded choice, we feel that *motivation* is the mechanism behind the decision to address healthcare inequities vs. address other areas. But the relationship between context, mechanism, and outcomes (CMO) is more complicated than a typical, unidirectional pathway. Our data suggest that the relationship between context, mechanism, and outcomes is bidirectional and that both negative and positive feedback may exist. For example, having a mentor may increase motivation to address healthcare inequities or being motivated to address healthcare inequities (e.g., wanting to “give back”) may help a physician find a mentor. Similarly, motivated individuals may seek out opportunities to work with “marginalized communities” or individuals who find themselves working with marginalized communities may become motivated to address inequities knowing that otherwise “patients tend to present again with the same problems a few days later” (and they may also be more likely to be surrounded by mentors). Despite the complicated interrelationship between context, mechanism, and outcome in this situation, we feel that the decision to become actively involved in addressing healthcare inequities associates with access to contextual facilitators (e.g. protected time, funding, and mentorship) and personal motivation, and that this motivation develops through prior experiences (e.g. working as a nurse near a First Nations reserve, volunteering internationally with health projects) and/or through direct interactions with inequities in clinical practice.

## Discussion

The Royal College of Physicians and Surgeons of Canada’s CanMEDS framework, describes a set of competencies Canadian physicians must possess to effectively care for their patients. One of its components is health advocacy, which is described as contributing to efforts to improve the health of patients, including “promoting health equity, whereby individuals and populations reach their full health potential without being disadvantaged by, for example, race, ethnicity, religion, gender, sexual orientation, age, social class, economic status, or level of education” [12]. Unfortunately, in our current healthcare system, many individuals do not “reach their full health potential”. All of the physicians that we interviewed as part of our study were aware of this fact and all felt that physicians should have a role in addressing the causes of this, but only some chose to become actively involved in initiatives to address healthcare inequities. This decision appears to be associated with experiences prior to medical training, through volunteer work or through personal experiences of having faced some form of inequity (e.g.: being immigrants to Canada). We postulate that being exposed to inequities (in healthcare or otherwise), motivated many of the AI physicians to seek out facilitators, mentors and system supports to help them address healthcare inequities.

These experiences do not have to have occurred prior to medical training, as demonstrated by one AI physician who saw healthcare inequities when they started practising at the Student Run Clinic, and consequently became motivated to do something about them. Conversely, physicians in the NAI group were less likely to have been exposed to health inequities prior to becoming physicians, and consequently were more likely to pursue other interests, despite facing similar barriers to involvement as AI physicians did.

Given how scant the literature is on this topic, a study that compares physicians who have been exposed to healthcare inequities, compared to physicians who have not, would shed light on the role of prior experiences in motivating physicians to tackle healthcare inequities.

Our study has some limitations that we should highlight. This was a single centre study in an urban Canadian centre, so our findings may not generalizable. We had a small sample size and, although our last two interviews did not yield any new themes, we cannot be confident that we reached thematic saturation given our sample size. We feel that further studies with larger sample sizes are needed to demonstrate thematic saturation and confirm or refute our findings. In addition, studies conducted at different centres in Canada, or in another healthcare system such as the United States, will help to determine the generalizability of our findings and perhaps identify other contextual factors and mechanisms. Once we have a better understanding of why some physicians choose to become involved in addressing healthcare inequities we can then explore ways of motivating more physicians and empowering them with contextual facilitators in the hope of collectively addressing healthcare inequities and allowing more individuals to “reach their full health potential”.

## Conclusion

As one of our physician colleagues, who also happened to be the Canadian Minister of Health at the time (Dr. Jane Philpott), reminded us: “because you are a doctor, society has granted you power and privilege, respect and responsibility. There is no better use of that power than to advocate on behalf of those who do not have the same opportunities” [2]. As physicians, we frequently witness the adverse effects of healthcare inequity and, collectively, there is a desire to address inequity. Hopefully, by improving our understanding of why some physicians choose to address inequities and identifying ways to increase the number of physicians that are motivated to address inequities, we can help more disadvantaged individuals overcome barriers to care.

## Appendix

### Interview Questions

Introductory statement:

I am conducting a research study for MDCN 440 – Applied Evidence Based Medicine (AEBM). AEBM is a course in our medical school, where we get dedicated time to engage in research work.

Under the supervision of Dr. McLaughlin, I am using this opportunity to learn how to conduct a research study. Through a series of interviews, I hope to explore the reasons and solutions for inequities that exist in our healthcare system.

The interview is confidential; there will be no identifying information attached to what you say today. Once the interview is completed, your responses will be anonymized and combined with responses from other interviewees. With that being said, you are free to decline to answer any of the questions I ask you today.

Since this is a learning opportunity for me, I will also ask you for any feedback you may have for me, at this end of this interview.

Do you have any questions before we begin?

Interview Questions:

- Are you aware of inequities in healthcare?
  i. How did you become aware of this?
- Have you given any thought as to why these inequities exist and how to overcome them?
- Can doctors have a role in addressing these inequities? If yes, how? If no, barriers?
- Should doctors have a role? Why? Why not?
- As a physician, there are many ways to spend your time, whether it’s seeing patients, conducting research, teaching, working in administration, among other things. Some of them may be related to working to address health inequities, of which there are also many ways of doing so. So, my next question is: ...
  Have you been involved in any initiatives, research projects, or other types of work to address a health inequity?
  i. What are some factors that enabled you to engage in this type of work (facilitators)?
  ii. What barriers have you encountered that have made it difficult for you to engage in this type of work?
- In your perspective, what is one of the greatest health inequities that needs to be addressed?
- Finally, I will like to ask a few questions to get some basic demographic information about you. Given our small sample size, we will not use this information for analysis; it is merely to describe some details of our study sample. Once again, you are free to decline to answer any of the questions I ask.
  i. Do you have a GFT or a major clinical position?
  ii. Have you been in practice < 10 years, 10–20, or > 20 years?
  iii. Do you identify as female, male, or other?

That concludes our interview today. Thank you so much for your time. Before we leave, do you have any questions for us? Do you any feedback for me?

## Abbreviations

AI: Actively involved
CMA: Canadian Medical Association
NAI: Not actively involved

## References

[1] Canadian Medical Association. CMA position statement: ensuring equitable access to care: strategies for governments, health care planners and the medical profession. In: Policies and research 2014. https://www.cma.ca/Assets/assets-library/document/en/advocacy/PD14-04-e.pdf. Accessed 21 July 2017.

[2] Picard A (2017). Health minister Jane Philpott urges physicians to use power, privilege to help Canada’s vulnerable.

[3] Barnett JC, Vornovitsky M. Health insurance coverage in the United States 2015. In United States Census Bureau Library. 2016; https://www.census.gov/library/publications/2016/demo/p60-257.html. Accessed 21 July 2017

[4] Government of Canada (2016). Canada's Health Care System. Health System and Services.

[5] McKeary M, Newbold B. Barriers to care: the challenges for Canadian refugees and their health care providers. J Refug Stud. 2010; 10.1093/jrs/feq038.

[6] Frankish CJ, Hwang SW, Quantz D, Hulchanski DJ, Campsie P, Chau SBY, Hwang SH, Paradis E (2009). The relationship between homelessness and health: an overview of research in Canada. Finding home: policy options for addressing homelessness in Canada. Toronto: Canadian observatory on homelessness.

[7] Peiris D, Brown A, Cass A. Addressing inequities in access to quality health Care for Indigenous people. Can Med Assoc J. 2008; 10.1503/cmaj.081445. 10.1503/cmaj.081445 PMC2572646 18981431

[8] Roos NP, Sullivan K, Walld R, MacWilliam L (2004). Potential savings from reducing inequalities in health. Canadian Journal of Public Health.

[9] Wong G, Westhorp G, Pawson R, Greenhalgh T. Realist synthesis. In: Rameses training materials. 2013. http://www.ramesesproject.org/media/Realist_reviews_training_materials.pdf. Accessed 30 July 2017.

[10] Bryant A, Charmaz K (2007). The SAGE handbook of grounded theory.

[11] Linsley P, Howard D, Owen S (2015). The construction of context-mechanisms-outcomes in realistic evaluation. Nurse researcher.

[12] Frank JR, Snell L, Sherbino J. CanMEDS 2015 Physician competency framework. In: Framework CanMEDS 2015. 2015. http://canmeds.royalcollege.ca/uploads/en/framework/CanMEDS%202015%20Framework_EN_Reduced.pdf. Accessed 15 December 2017.

